# Accurate diagnosis of prostate cancer using logistic regression

**DOI:** 10.1515/med-2021-0238

**Published:** 2021-03-24

**Authors:** Arash Hooshmand

**Affiliations:** Department of Biomedical Engineering and Health Systems, School of Engineering Sciences in Chemistry, Biotechnology and Health, KTH Royal Institute of Technology, 11428 Stockholm, Sweden

**Keywords:** machine learning, prostate cancer, diagnosis, transcriptome, RNA sequencing, high throughput technologies, logistic regression, classification

## Abstract

A new logistic regression-based method to distinguish between cancerous and noncancerous RNA genomic data is developed and tested with 100% precision on 595 healthy and cancerous prostate samples. A logistic regression system is developed and trained using whole-exome sequencing data at a high-level, i.e., normalized quantification of RNAs obtained from 495 prostate cancer samples from The Cancer Genome Atlas and 100 healthy samples from the Genotype-Tissue Expression project. We could show that both sensitivity and specificity of the method in the classification of cancerous and noncancerous cells are perfectly 100%.

## 1 Introduction

Prostate cancer is one of the severe cancers in men. According to the US cancer statistics report for 2020, there are estimated 191,930 new cases of prostate cancer and 33,330 deaths because of it, and the importance of early diagnosis has repeatedly been emphasized [1]. Biologists have discovered many genes that are involved in specific cancers; for example, BRCA1 in breast cancer [2] and STAT3 in prostate cancer [3]. In diagnosis and cancer identification, histological examination is used as gold standard but it is a slow process and needs technical experts and suffers from large amount of variations among observers. In recent years, thanks to high throughput Omics technologies, we are no longer missing data but need novel methods and techniques to handle and analyze them; thus, bioinformatics and computers have found a solid ground to contribute in life sciences. One of the most applicable approaches to benefit from computer science in physiology and medicine is utilization of artificial intelligence to extract knowledge by computers out of big data generated by Omics technologies [4]. In this work, we have developed a logistic regression (LGR) system using general new generation of RNA Seq. data that can detect any prostate cancer, and hence will decrease the risk of mortality by correct diagnosis. The Omics technologies and their corresponding big data analysis tools are developing fast and getting cheaper and more widespread all the time. Currently, the third generation of sequencing methods such as quantum sequencing [5], nanopore sequencing [6], and single-molecule real-time sequencing [7] are making it possible even today for the wealthy people to benefit from expensive analyses, and if the current trend in advancements continues, it will not be a long way left to have commonplace analytical tools and services in each hospital and city. The advantage of machine learning is that as it gets more and more samples, its training would be more matured and more robust; therefore, there is a hope that the 100% accuracy that is achieved by a modest amount of data can be stabilized in the future when many patients and healthy people samples are fed to the system.

Computational techniques and tools are rapidly opening their positions in medical and pharmaceutical sciences too [8]. Different methods have been developed and tested in the last few decades and have returned great results in different fields of medicine including but not limited to cancer identification [9]. In this work, we have come up with a novel approach of applying LGR for cancer detection that is effective and robust. Using our method, cancerous tissue can correctly be identified, thus providing an opportunity to be controlled on time. This approach also offers a new direction for disease diagnosis while providing a new method to predict traits based on genomic information.

## 2 Methods

In this project, we have used LGR algorithm from Sci-Kit Learn on 495 samples from The Cancer Genome Atlas (TCGA) research network and 100 samples of the Genotype-Tissue Expression (GTEx) project portal and directly fed the genome data to the machine to do heavy statistical calculations on our high dimensional data. The different parts of the method are clarified below. We use all the available data at the time of accessing the databases and have not ignored any sample.

### 2.1 Binary LGR

The LGR is a group of statistical techniques that aim to test hypotheses or causal relationships when the dependent variable is nominal.

Despite its name, it is not an algorithm applied in regression problems, in which continuous values are dealt with, but it is a method for classification problems, in which a binary value, i.e., either 0 or 1 is obtained. For example, a classification problem is to identify if a given tumor is malignant or benign. With the LGR, the relationship between the dependent variable, i.e., the statement to be predicted, with one or more independent variables, i.e., the set of features available for the model is determined. To do this, it uses a logistic function that determines the probability of the dependent variable. As previously mentioned, what is sought in these problems is a classification, so the probability must be translated into binary values for which a threshold value is used. If the probability values were above the threshold value, the statement is true and *vice versa*. Generally, this value is 0.5, although it can be increased or decreased to manage the number of false positives or false negatives [10].

In supervised classification methods the input data, usually seen as p points, are viewed as a p-dimensional vector (an array or ordered list of p numbers). Then the classifiers are more or less based on similar criteria, e.g., in the Bayesian classifiers, the classifier looks for a hyper surface that maximizes the likelihood of drawing the sample, or in SVMs, it looks for a hyperplane that optimally separates the points of one class from the other, which eventually could have been previously projected to a higher dimensional space. The LGR is a generalization of logits to distinguish samples that belong to one of the two different classes; hence, it is usually called binary LGR.

### 2.2 Feature selection

There are wrong perceptions in the computer science community about life science data that have prevented potential achievements, for instance, one is about the number of features [11] such as “it is obviously impractical to select all of the genes because mass dimensions will increase the computation cost.” As a result, researchers usually try to reduce the assumed computational costs allegedly brought about by highly redundant dimensions and select a subset of features, i.e., genes to reduce the number of features and dimensions [12]. A strength point of our work is that we gave all the data corresponding to the whole-exome sequencing as feature inputs to the logistic regressor at once and it returned almost perfect results quickly and precisely. We thought of 19,627 different genes not as too many features but as different pixels of a less than 141 × 141 pixel photo, in which there are correlated pixels too, and it was a very light task for the machine to analyze such a low-resolution image and it took only seconds to classify the cancerous and noncancerous cells 100% precisely.

### 2.3 Model settings and evaluation

We have used LGR classifier also known as Logit or MaxEnt classifier from Scikit-Learn 0.23.1 with its default settings. Model evaluation produces measures to approximate a classifier’s reliability. To distinguish between cancerous and noncancerous cells, as it is a binary classification, we use accuracy, precision, specificity, sensitivity, f1 score, several averaging techniques, and receiver operating characteristic curve to evaluate the model. We, indeed, use Sci-kit Learn Metrics Classification Report that returns precision, recall, and f1 score for each of two classes. In binary classification, recall of the positive class is called “sensitivity,” and recall of the negative class is “specificity.” In what follows, the terms and derivations from confusion matrix such as accuracy, specificity, sensitivity, and f1 score are given to review and compare:

Condition positive (P): the number of real positive cases in the data
Condition negative (N): the number of real negative cases in the data
True positive (TP) or hit
True negative (TN) or correct rejection
False positive (FP), false alarm, or type I error
False negative (FN), miss, or type II error

Sensitivity, recall, hit rate, or true-positive rate (TPR):

(1) \text{TPR}=\text{TP}/P=\text{TP}/(\text{TP}+\text{FN})=1-\text{FNR}.]

Specificity, selectivity, or true-negative rate (TNR):

(2) \text{TNR}=\text{TN}/N=\text{TN}/(\text{TN}+\text{FP})=1-\text{FPR}.]

Precision or positive predictive value (PPV) is the ratio of the correctly labeled samples by our program to all labeled ones in reality.

(3) \text{PPV}=\text{TP}/(\text{TP}+\text{FP})=1-\text{FDR}.]

Precision can be calculated only for the positive class, i.e., class 1 that shows cancer or can be evaluated for each one of the two classes independently treating each class as it is the positive class at time, and the latter is done in Sci-kit Learn Metrics Classification Report as shown in Table 1.

**Table 1:** Classification report

Summary	Precision	Recall	f1 score	Support
Class 0	1.00	1.00	1.00	9
Class 1	1.00	1.00	1.00	51
Micro avg.	1.00	1.00	1.00	60
Macro avg.	1.00	1.00	1.00	60
Weighted avg.	1.00	1.00	1.00	60

Negative predictive value (NPV):

(4) \text{NPV}=\text{TN}/(\text{TN}+\text{FN})=1-\text{FOR}.]

Miss rate or false-negative rate (FNR):

(5) \text{FNR}=\text{FN}/P=\text{FN}/(\text{FN}+\text{TP})=1-\text{TPR}.]

Fall-out or false-positive rate (FPR):

(6) \text{FPR}=\text{FP}/N=\text{FP}/(\text{FP}+\text{TN})=1-\text{TNR}.]

False discovery rate (FDR):

(7) \text{FDR}=\text{FP}/(\text{FP}+\text{TP})=1-\text{PPV}.]

False omission rate (FOR):

(8) \text{FOR}=\text{FN}/(\text{FN}+\text{TN})=1-\text{NPV}.]

Accuracy (ACC):

(9) \text{ACC}=(\text{TP}+\text{TN})/(\text{T}+\text{N})\hspace{2.2em}=(\text{TP}+\text{TN})/(\text{TP}+\text{TN}+\text{FP}+\text{FN}).]

The harmonic mean of precision and sensitivity or f1 score (F1):

(10) \text{F}1=2\cdot \text{PPV}\cdot \text{TPR}/(\text{PPV}+\text{TPR})=2\cdot \text{TP}/(2\cdot \text{TP}+\text{FP}+\text{FN}).]

As we are using Sci-kit Learn Metrics Classification Report to show the results as shown in Table 1, we also describe the meaning of micro avg, macro avg, and weighted avg. used in the report: Micro-average of precision (MIAP):

(11) \text{MIAP}=(\text{TP}1+\text{TP}2)/(\text{TP}1+\text{TP}2+\text{FP}1+\text{FP}2).]

Micro-average of recall (MIAR):

(12) \text{MIAR}=(\text{TP}1+\text{TP}2)/(\text{TP}1+\text{TP}2+\text{FN}1+\text{FN}2).]

Micro-average of f-score (MIAF) would be the harmonic mean of the two numbers above.

(13) \text{MIAF}=2\cdot \text{MIAP}\cdot \text{MIAR}/(\text{MIAP}+\text{MIAR}).]

Macro-average of precision (MAAP):

(14) \text{MAAP}=(\text{Precision}\hspace{.5em}1+\text{Precision}\hspace{.5em}2)/2.]

Macro-average of recall (MAAR):

(15) \text{MAAR}=(\text{Recall}\hspace{.5em}1+\text{Recall}\hspace{.5em}2)/2.]

Macro-average of f-score (MAAF) would be the harmonic mean of the two numbers above.

(16) \text{MAAF}=2\cdot \text{MAAP}\cdot \text{MAAR}/(\text{MAAP}+\text{MAAR}).]

Macro-average method is suitable to know how the system performs overall across different sets of data but should not be considered in any specific decision-making because it calculates metrics for each label and finds their unweighted mean, i.e., it does not take label imbalance into account, while in our case, the labels are highly imbalanced, i.e., 495 vs 100. On the contrary, micro-average is a useful tool and returns measures for decision-making especially when datasets vary in size because it calculates metrics globally by counting the total true-positives, false-negatives, and false-positives. Finally, weighted-average, according to Sci-kit Learn documentation on f1-score metrics, calculates metrics for each label and finds their average weighted by support (the number of true instances for each label). This alters “macro” to account for label imbalance; consequently, it can result in an F-score that is not between precision and recall.

## 3 Results

**Figure 1:**
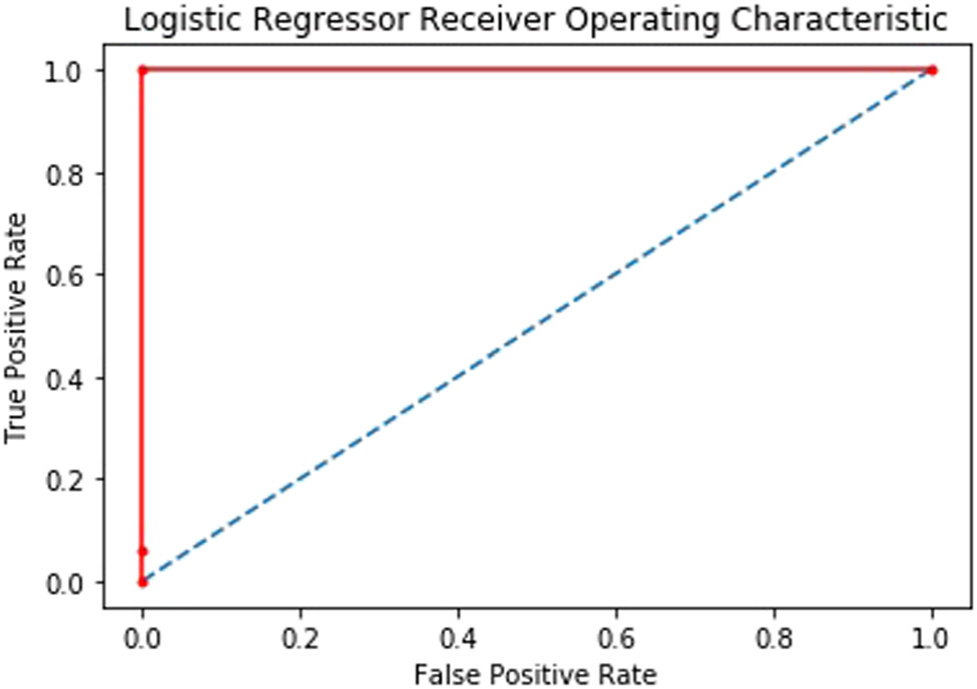
ROC curve of LGR classifier performance in distinguishing cancerous and noncancerous prostate cells.

Genomic variation files of 595 samples including healthy people (100 individuals) and cancer patients (495 individuals) were obtained from the GTEx Project and the TCGA online database. The binary classification results of cancerous and noncancerous samples were great because the system can detect all cancerous and noncancerous samples correctly and as seen in the classification report shown in Table 1, the performance of the classifier is perfect with accuracy and precision of 100% and sensitivity and specificity of 1. In this classification, not only the accuracy is 100% but also the receiver operating characteristic’s area under curve (ROC AUC) from prediction scores also would be 1 as seen in Figure 1.

## 4 Discussion

The classifier did its task perfectly with no error, at least on our available data. There are yet some aspects to reflect on. Although most TCGA prostate cancer (PRAD) comprise white men’s samples, they have considered human variations to contain samples of different races and groups as well to represent the US demographic information fairly. As our method classifies all cancerous and non-cancer samples correctly using the information available in genomic variation, it can mean that the genetic signatures of cancer are detected universally without the need to consider racial or sexual differences.

Our work provided a new approach in application of computers using medical data that resulted in excellent classification between cancerous and noncancerous cells of the prostate. In this work, we did not reduce the dimension of input data and left all the statistical analysis to the computer, and it could do its job very well and distinguished the cancerous samples from healthy cells almost perfectly. We even did not need to balance the number of samples of each class and it shows that the difference between two classes is so much that providing hundreds of samples enables the machine to distinguish between two categories containing 495 and 100 samples perfectly. It is also useful to consider the fact that TCGA and GTEx data are not perfect and there are several rows of missing data for some of gene quantities in some samples, yet the data provided by these two projects are fairly clean and reliable and it was enough for our classifier to be able to do its classification 100% correctly. This system is trained now to receive any new person’s RNA-seq data and recognize if the patient’s prostate is cancerous or not. The limitation of our model is that it needs future collaboration with both hospitals and well-equipped laboratories and also needs the whole genome data of samples from the organ, and the involving labs should follow the same protocols to obtain the transcriptomics data. Therefore, we cannot add training data from other sources and databases to include as many samples as we want. Fortunately, we do not need to do it because our data have been enough to train the system and achieve perfect classification ability. Furthermore, an advantage of our approach is that we have used a classic interpretable method that is based on statistics, unlike other works such as Sun et al. [13] who have used complex neural networks that act as a black box and are not interpretable. Nevertheless, obtaining the whole-exome sequencing data of 19,627 genes as done by GTEx and TCGA on samples obtained from people’s prostates is at research level and is not yet a cheap procedure or common practice for general hospitals. However, the New Generation RNA-seq protocols followed by GTEx and TCGA are well known and standard, and as technologies are developed rapidly, they are continuously getting cheaper and more practical than before. Meanwhile, the next topic of research can be finding suitable biomarkers in the blood that can detect healthy people and patients only by their blood tests.
